# An Investigation on Fatigue Resistance of Notched Long Fiber-Reinforced Composite Materials

**DOI:** 10.3390/polym14040822

**Published:** 2022-02-21

**Authors:** Lili Cao, Qipeng Li, Zhongwang Niu, Yuanyuan Zheng

**Affiliations:** 1 School of Mechanical and Energy Engineering, Zhejiang University of Science and Technology, Hangzhou 310023, China; 112016@zust.edu.cn; 2 Ningbo Sunny Automotive Optech Co., Ltd., Ningbo 315400, China; czniuzw@sunnyoptical.com

**Keywords:** notched fiber, composite material, tensile strength, fatigue property

## Abstract

A new type of specimen is proposed for further research on the structure of glass-fiber-reinforced resin matrix composite lamina, which holds the potential to significantly improve the fatigue property of materials while having limited effect on the tensile strength. Herein, the fatigue life, based on the monotonic tensile test, was simulated utilizing ANSYS and nCode analysis software. The results show that the tensile strength of the local notched fiber specimens is slightly lower than that of the continuous long-fiber specimens. However, when extending the notches’ longitudinal distance, the impact to tensile strength becomes smaller and smaller. The results show that, when the longitudinal distance of the notched fiber is greater than 80 mm, the reduction in tensile strength is less than 0.65%. At the same time, the fatigue property of the specimens is improved considerably. It has been found in this experiment that when the notches’ longitudinal distance is 100 mm, the notches’ length is 1.5 mm, and the notches’ width is 1.75 mm, the fatigue cycles number of the specimens reaches 126,000 cycles, which is about 180% higher than that of the 0-0 type long fiber specimens without notches. This investigation provides a robust foundation and is a compelling basis for further exploration of new fatigue specimens.

## 1. Introduction

With the development of science and technology, the demand for high-strength and light-weight new materials is increasing, and new materials and technologies are emerging. Fiber-reinforced resin matrix composites are widely used in aerospace, military, mechanical, and other fields because of their excellent comprehensive properties [1,2], such as high specific strength, light weight, and strong structural designability. Meanwhile, their consumption is increasing significantly [3]. With the continuous improvement in technology, the strength of materials is also improving [4,5]. However, when the structure is subjected to cycling load, it often leads to fatigue failure. This means, on one hand, that the strength of the material is increased, but on the other hand the fatigue life is reduced. This has become one of the “bottleneck” problems in the development of composite materials.

In recent years, the relationship between the strength and fatigue properties of fiber-reinforced composites has been attracting the attention of many material scientists. For example, B.-L.MA et al. [6] studied the effects of humid and hot environments on the fatigue reliability life of carbon-fiber composite laminates. The results show that the change trend in the stiffness degradation curve of the test specimens was consistent: as the reduction in stiffness increases, the fatigue limit decreases by about 6%. The fatigue damage mode is similar, but the damage degree is intensified under the same number of the fatigue cycle. N. H. Padmaraj et al. [7] studied the fatigue behavior of glass/epoxy quasi isotropic laminated composites under different aging conditions. The results show that the fatigue damage expansion depends on aging conditions and moisture content. The fatigue property degradation is due to the multiple cracks on the surface of the fiber and matrix decomposition. N. Velmurugan et al. [8] studied the effect of silanized reinforcements on the viscoelasticity and fatigue property of E-glass fiber-reinforced epoxy composites. The results show that the fatigue and viscoelasticity properties of the composite can be improved by adding microrubber into the epoxy resin, and the addition of silicon carbide can improve the property of the composite significantly. The maximum fatigue life cycle of SiC and microrubber composites with 1% and 10% volume fraction is 12,941, and the storage modulus is 7.8 GPa. D. Mei et al. [9] used vacuum-assisted molding to prepare glass fiber-reinforced composites with different resin content and layers to explore the effect of stress amplitude on the fatigue property. The results show that the fatigue property of the material decreases with the increase in the number of glass fiber layers, and the fatigue life at 40% stress amplitude is about 4–5 times that at 50% stress amplitude. J. Xu et al. [10] studied the tensile fatigue property of carbon glass hybrid fiber-reinforced composites. The experimental data show that the fatigue property of carbon and glass hybrid fabric-reinforced composite is in line with the linear mixing law, but the error is relatively large under low cycle fatigue. X. Zhang et al. [11] studied the tensile fatigue property of the orthogonal laminated plate of carbon fiber composite after moisture absorption. The results show that saturated moisture absorption has a great influence on the tensile fatigue property of the orthogonal laminated plate. The tensile fatigue life of the composite plate after moisture absorption is obviously lower than that of the dry state, and the slope of the S-N curve is low. The fiber damage initiation and expansion of the composite plate after moisture absorption are also different from the dry state.

At present, although scientists have done much research on fiber-reinforced resin matrix composites, they mainly focus on the establishment of a fatigue damage model of composites [12,13,14], finite element simulation of structural fatigue [15,16,17], and analysis of mechanical properties and fatigue properties [10,18,19]. There are two main applications of fiber-reinforced composites—long fiber-reinforced composites and short fiber-reinforced composites—but each possesses disadvantages. Long fiber-reinforced composites have high strength but poor toughness and fatigue properties. Short fiber-reinforced composites have good toughness and fatigue property but low strength [20,21,22]. That is to say, it is still impossible to effectively improve the fatigue property of the material while ensuring the strength of the material to meet the requirements, especially of stress fatigue and strain fatigue under different conditions. In this paper, the structure of glass fiber-reinforced resin matrix composite laminates is studied in detail, and a new type of specimen is proposed, which can significantly improve the fatigue property with limited impact to the tensile strength.

## 2. Structural Design of New Fiber-Reinforced Composites

**Figure 1:**
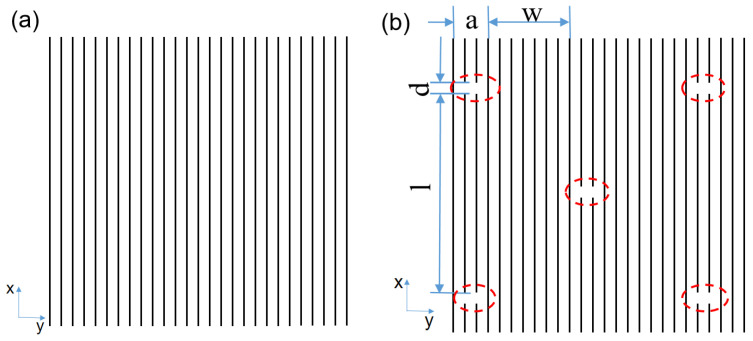
Traditional structural design and innovative structural design. (**a**) Traditional monolayer with complete long fibers (**b**) Interlaced notched monolayer. Note: l is the longitudinal distance between two adjacent notches, w is the lateral distance between two adjacent notches, a is the notches length, and d is the longitudinal distance of the notches.

In this paper, the innovative structure of glass fiber-reinforced resin matrix composite laminates is designed. Different from the traditional structure, a monolayer with interlaced notches is shown in Figure 1. Additionally, five kinds of glass fiber-reinforced resin matrix composite laminates with different structure were prepared, which paves the way for the subsequent tensile test, fatigue life simulation, and conclusion verification of glass fiber (carbon fiber, etc.) reinforced resin matrix composite specimens.

First, a group of untreated glass fiber-reinforced resin matrix composite monolayers were taken as the standard comparison group, marked as 0-0; the mechanical properties of the materials are shown in Table 1. The remaining groups of monolayers were subjected to fiber cutting treatment, as shown in Table 2.

Then, five monolayers in the same direction were superposed and extruded by a plate-type laminating machine (equipment name is Meyer, model is kfk-x 1900) to form a glass fiber-reinforced resin matrix composite laminate. The ply angle between each monolayer was 0. The second and fourth layers are the same type of monolayers, and the first, third, and fifth layers were 0-0-type monolayers. Finally, five kinds of glass fiber-reinforced resin matrix composite laminates (0-0, 4-2, 6-2, 8-1 and 8-2) were prepared.

The fiber of the glass fiber-reinforced resin matrix composite monolayers was cut off. By changing the longitudinal distance of the notches, the monolayer with different structure was designed. Five kinds of glass fiber-reinforced resin matrix composite laminates with different structure were prepared by rolling monolayers onto a plate-type laminating machine, which paved the way for subsequent tensile test and fatigue life simulation.

## 3. Tensile Test and Analysis

Tensile test is one of the most important and common methods to test the mechanical properties of materials. The basic mechanical properties data such as yield strength, tensile strength, elastic modulus, and plastic strain ratio can be obtained by tensile test. These data are very important for the research and development of new materials, the control of product quality, and the evaluation of equipment safety. Five kinds of glass fiber-reinforced resin matrix composite laminates with different structures were prepared as explained in the previous section. The specimens required for tensile test were prepared according to the standard GB/T 3354-2014 [23], the tensile test was completed, and the results were analyzed.

The raw material of this experiment is glass fiber-reinforced composite single-layer plate (450 mm × 450 mm × 0.25 mm). The plate-type compound machine uses the upper and lower conveyor belts to transmit pressure and integrates the contact heating and cooling system. The materials between the upper and lower conveyor belts were evenly heated and cooled to normal temperature before leaving the conveyor belt. The single-layer board and flat-panel compound machine were provided by Zhejiang Huajiang Technology Co., Ltd. The tensile testing machine model was a wdw-100 microcomputer-controlled electronic universal testing machine, which was provided by Zhejiang University of Science and Technology.

### 3.1. Specimens

**Figure 2:**
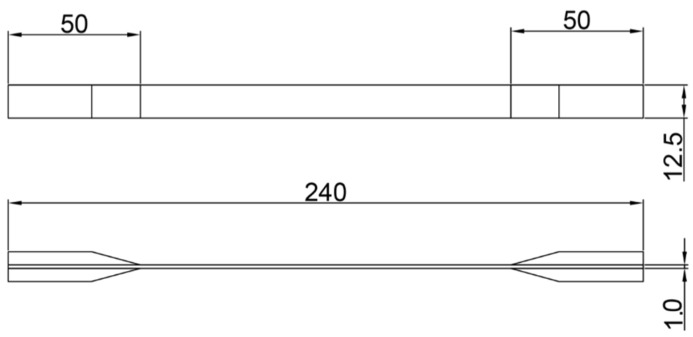
Basic dimensions of tensile specimen (refer to national standard GB/T 3354-2014) [23].

The thickness of the specimens was 1 mm, the length was 240 mm (note: to ensure the effective length, it can be adjusted accordingly), and the width was 12.5 mm. The two ends of the specimens were the clamping ends of the fixture, and the middle part was the test area. The structure is shown in Figure 2.

### 3.2. Tensile Test and Result Analysis

**Figure 3:**
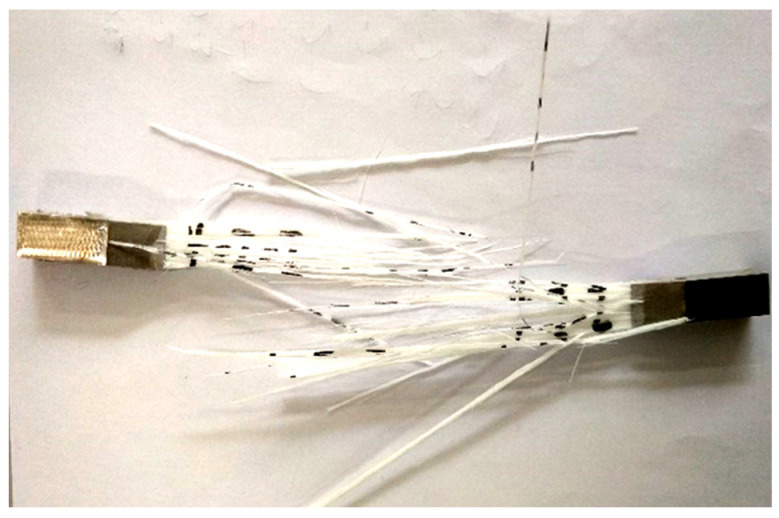
Fractured specimen.

**Figure 4:**
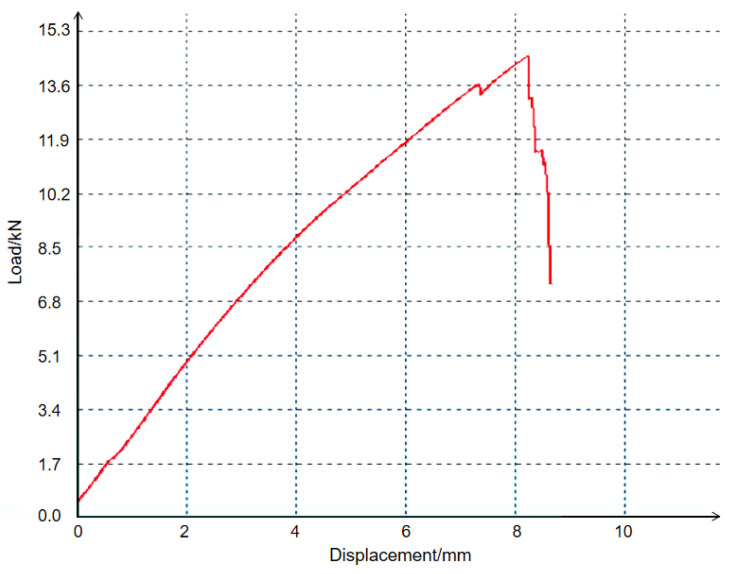
Tensile curve.

The tensile test of the specimens was carried out by the microcomputer controlled electronic universal testing machine (model is WDW-100). The fracture type 4-2 specimens and its tensile curve are shown in Figure 3 and Figure 4. Ten specimens were made for each type (note: if there is stress concentration at the chuck, the measured results are regarded as invalid), and then the average value was taken.

It can be seen from Figure 3 that the fracture mode of the specimen was tearing. The reason is that the fiber was the main load-bearing part and the matrix played the role of skeleton. When the stress exceeded the maximum stress that the specimen can bear, the local fibers broke, and these breaks gradually expanded to other fibers. Therefore, the tensile curve had a notched decline. Because the shear force between the fiber and the resin matrix exceeded the interfacial bonding strength, the fiber and the resin matrix showed tearing failure. In Figure 4, the longitudinal axis is Displacement (mm) and the vertical axis is Load (kN). The curve increases linearly at the beginning, and when the tensile force reaches 10.6 kN, the curve begins to slow down until it disappears. During the test, with the increase in tensile force, some of the fiber layers of the specimen broke. Because the other fiber layers of the specimen did not break, it could still bear a certain tensile force. Then, the other fiber layers broke one after another, and the loading curve decreased slowly until all the fiber layers broke. Finally, when the bond strength between the resin matrix and the fiber could not bear the shear force, complete fracture occurred. The tensile results are shown in Table 3.

From the test results (Table 3), it can be seen that the tensile strength of the 0-0-type specimen is the highest. The tensile strength of 4-2, 6-2, 8-1, and 8-2 specimens with prefabricated notches decreased slightly. The most influential one was the 4-2 specimen (the longitudinal distance of notches is 40 mm), in which the tensile strength decreased by 9.43%; the smallest influence was 8-2 specimen (the longitudinal distance of notches is 80 mm), in which the tensile strength decreased only by 0.65% compared with the specimens without notches. In conclusion, with the increase in the longitudinal distance of the notches, the influence degree of the tensile strength is significantly reduced. In addition, the notch state of two adjacent lines was compared. The results show that the tensile strength of the type 8-2 specimen (in which the longitudinal distance of the notched fiber was 80 mm and the notched fiber state of two adjacent lines was interlaced) was better than that of type 8-1 specimen (in which the longitudinal distance of the notched fiber was 80 mm and the notched fiber state of two adjacent lines was parallel). Therefore, the tensile strength of specimens with interlaced notches was significantly higher than that of specimens with parallel notches.

## 4. Fatigue Life Simulation and Discussion

Fatigue tests are a very useful type of research work in material science. However, due to its time-consuming, labor-consuming nature and other reasons, it is not convenient to directly carry out a large number of in-depth experimental research studies. Fortunately, with the study of finite element theory and fatigue fracture analysis method in recent years, as well as the rapid development of computer technology, finite element and fatigue analysis software has appeared, such as ANSYS and nCode. ANSYS was used for finite element analysis of the specimen. Then, the analysis results were imported into nCode for fatigue life simulation, and the results were studied.

ANSYS ncode DesignLife is a fatigue analysis module fully integrated into the ANSYS Workbench platform. It is one of the most powerful software in the fatigue field. ANSYS ncode DesignLife comes with fatigue performance parameters of more than 200 materials, including a variety of composite and metal materials, which is very convenient to use. The calculation steps can be divided into importing finite element analysis results, adding load spectrum, defining material properties, and solving calculation and fatigue result evaluation.

Firstly, the stress–strain analysis of the specimen was carried out by ANSYS. Glass fiber-reinforced epoxy resin composite was used, and the material parameters were modified.

In order to facilitate the later fatigue numerical simulation, the tension was set to 4059 N. In order to improve the calculation speed and accuracy, the grid division density was 1 mm, and the grid division density near the notch area of the specimen was 0.2 mm. The division method is tetrahedral division. In this experiment, an incision was made on the single-layer plate, but when preparing the laminated plate, the resin melted and bonded the single-layer plate together. Therefore, the notch on the test piece was a not completely disconnected but a viscous connection. The two ends of the notch of the specimen were springs, and the spring stiffness was the elastic modulus of the resin. The weak spring was opened to counteract the small force deviation at both ends of the specimen due to accuracy problems. The finite element analysis results are shown in the figures.

In order to analyze the influence of different structures on the fatigue performance, new specimen types were added to the virtual part. The software simulates the fatigue cycle number of the prepared type specimen, further simulates the E-N curve and the fatigue cycle number of other four types of specimens (9-2, 10-2, 11-2, 12-2) according to the predicted ultimate tensile strength, and compares the simulation results.

The structural design of single-layer plate for other four types (9-2, 10-2, 11-2, and 12-2) of test specimens is shown in Table 4.

**Figure 5:**
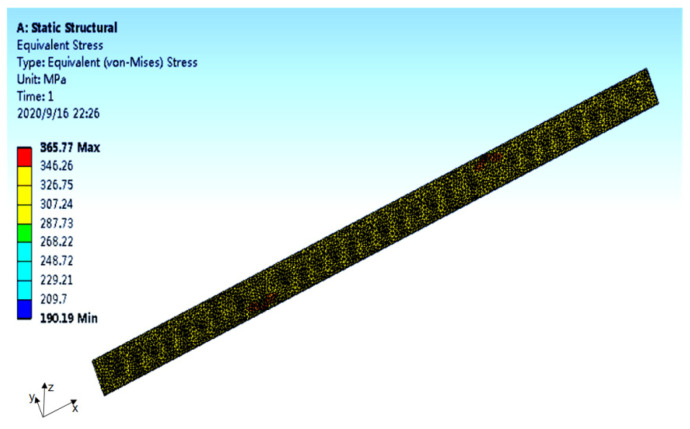
The finite element analysis results of the specimen stress (10-2).

**Figure 6:**
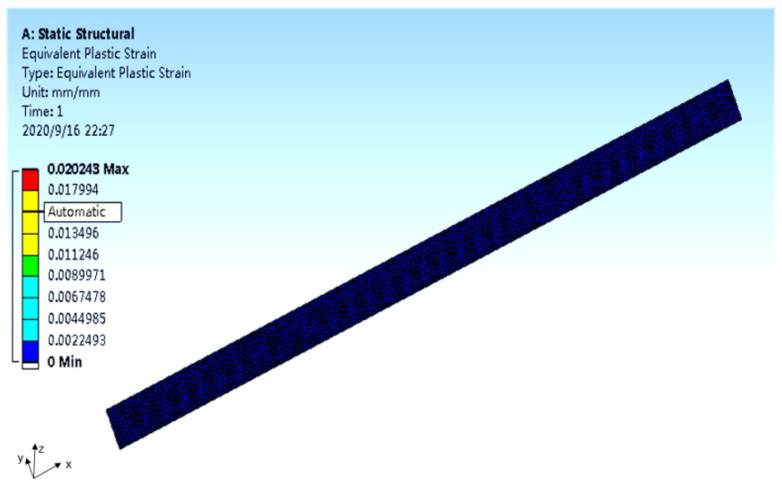
The finite element analysis results of the specimen strain (10-2).

The finite element analysis results of the specimen stress and strain are shown in Figure 5 and Figure 6 (take the 10-2 specimen as an example).

Results can be found in Figure 5. The stress at the notch was the highest, reaching 365.77 MPa. With the increase in distance from the notch, the stress decreased gradually. The stress far from the incision was the lowest, at only 190.19 MPa. There was a large gap between the two values, indicating that there was a concentration of stress at the incision. As can be seen from Figure 6, under the influence of stress concentration, the strain at the notch was the highest, reaching 0.020243 mm/mm. The strain decreased with the increase in the distance from the notch. The strain was zero at a distance from the incision.

**Figure 7:**
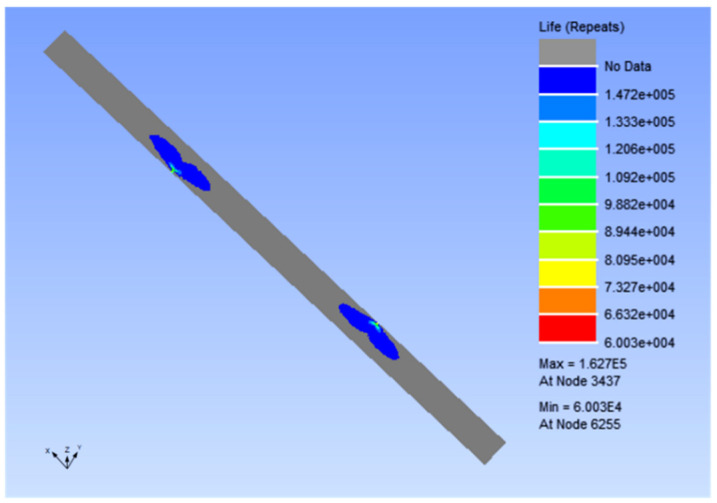
Strain fatigue numerical simulation results. Note: *x*-axis is the length direction; *y*-axis is the width direction, and *z*-axis is the normal direction of the unit.

Then, the analysis results were imported into nCode for fatigue life simulation. Due to the existence of notches on the specimens, the common strain fatigue (such as pressure vessel) was used for simulation, which ensured that the calculation software can accurately measure the fatigue data even if there is stress concentration. When using nCode to simulate strain fatigue, the two most important factors are the setting of material E-N curve and the selection of the load spectrum. Since there are no ready-made fatigue test data for reference, the standard E-N curve can only be estimated from the ultimate tensile strength of the material and then modified. The maximum tensile force was 60% of the ultimate tensile strength of the 0-0 specimen, which was 7.38 kN. The load spectrum was sinusoidal. The stress ratio R was 0.1. Additionally, the loading frequency was 10Hz. The numerical simulation results of strain fatigue of type 10-2 specimen are shown in Figure 7.

As can be seen from Figure 7, the number of fatigue cycles at the notch is the lowest, at only 60,030 cycles. The number of fatigue cycles in the sector outside the notch gradually increased to 147,200 cycles. If it was far away from the notch, the specimen was not damaged due to fatigue.

### 4.1. Effect of Notches Longitudinal Distance on Fatigue Property

**Figure 8:**
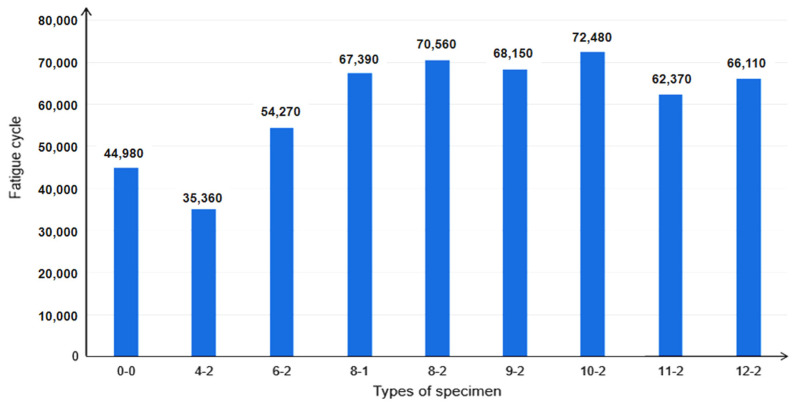
Effect of notches longitudinal distance on fatigue property.

The fatigue cycles of all types of specimens are simulated. The fatigue cycles of 9-2, 10-2, 11-2, and 12-2 specimens are simulated according to the predicted ultimate tensile strength, as shown in Figure 8.

It can be seen from Figure 8 that the fatigue cycle number of the type 4-2 specimen is lower than that of other types specimens, which is due to the too-small longitudinal distance between two adjacent lines’ notches. When the long fiber was cut into short fibers, the tensile strength decreased noticeably. Additionally, the ratio of stress to ultimate tensile strength was too large in the fatigue simulation, which led to a decrease in the fatigue property. Compared with 0-0 long fiber specimens without notches, other types of specimens showed improvement. Because 0-0 type specimens had high tensile strength and poor toughness, the number of fatigue cycles was not high. While the toughness of specimens with local fiber cut was improved under the premise of limited adjustment of tensile strength, the number of fatigue cycles showed a significant improvement. The fatigue cycles number of 10-2 type specimens reached the maximum value of 72,480 cycles, which was about 61% higher than that of 0-0 type long fiber specimens without notches. The fatigue cycle number of 11-2 and 12-2 type specimens was slightly lower than that of 10-2 type specimens and tended to be stable. This indicates that when the longitudinal distance of notches is more than 100 mm, the effect of the longitudinal distance of notches on the fatigue cycle number can be ignored. Furthermore, the fatigue cycle number of 8-2 (the notches are interlaced) type specimens is obviously higher than that of 8-1 (the notches are parallel) type specimens. It can be seen that the fatigue cycle number of the specimens with interlaced notches between two adjacent lines was higher than that of the specimens with parallel notches between two adjacent lines

**Figure 9:**
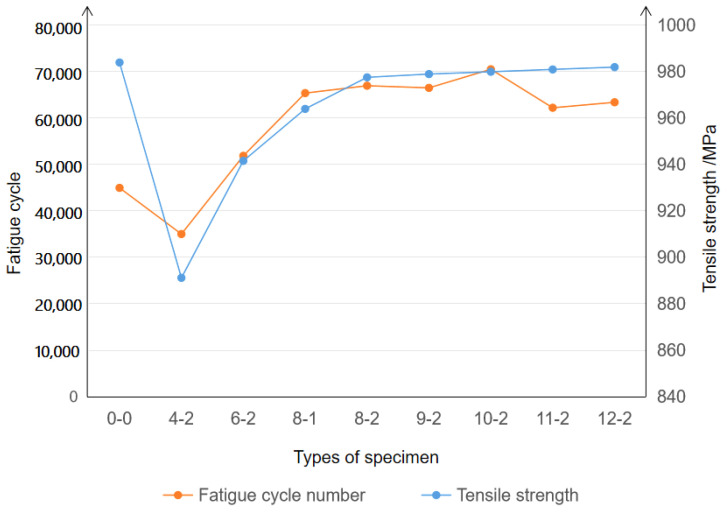
Strength-fatigue comprehensive mechanical properties.

Figure 9 shows the comparison of strength-fatigue comprehensive mechanical properties of glass fiber-reinforced resin matrix composites. The tensile strength of 0-0 specimens was the highest, but the fatigue property was poor, and the strength-fatigue comprehensive mechanical properties were general. The tensile strength of 8-2, 9-2, 10-2, 11-2, and 12-2 specimens was slightly lower than that of 0-0 specimens, but the fatigue property was greatly improved, and the strength-fatigue comprehensive mechanical properties were better. The mechanical properties of 10-2 specimen were the best.

Figure 9 shows the comparison of strength-fatigue comprehensive mechanical properties of glass fiber-reinforced resin matrix composites. The tensile strength of 0-0 specimens was the highest, but the fatigue property was poor, and the strength-fatigue comprehensive mechanical properties were at a normal level. The tensile strength of 8-2, 9-2, 10-2, 11-2, and 12-2 specimens was slightly lower than that of 0-0 specimens, but the fatigue property is greatly improved, and the strength-fatigue comprehensive mechanical properties were better. The mechanical properties of 10-2 specimen were the best.

### 4.2. Effect of Notches Length on Fatigue Property

**Figure 10:**
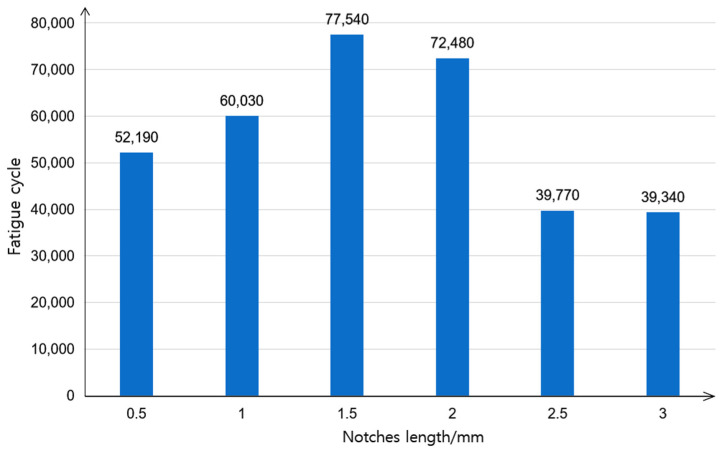
Effect of notches length on the fatigue property.

Through the research on the influence of different notches’ longitudinal distance on fatigue properties, it is known that when the notches’ longitudinal distance is 100 mm, the fatigue property of the specimens is the best. Additionally, the comprehensive strength-fatigue mechanical properties are the best. Therefore, under the condition that the notches’ longitudinal distance is 100 mm, the influence of different notch lengths on the fatigue property of the specimens is explored by changing the notches’ lengths. The final simulation results are shown in Figure 10.

It can be seen from Figure 10 that with the increase in the notches’ length, the fatigue cycle number of the specimens first increased and then decreased. When the notches length was 1.5 mm, the fatigue cycles number of the specimens reached a maximum value of 77,540 cycles, which is about 72% higher than that of the 0-0 type long fiber specimens without notches. When the notches length was less than 1.5 mm, with the increase in the notches’ length, the tensile strength of the specimens with local fiber cut decreased continuously in the effective range. Additionally, the toughness increased, and the fatigue cycle number increased. When the notches length was greater than 1.5 mm, the tensile strength of the specimens decreased rapidly with the increase in the notches’ length. Because the ratio of the stress to the ultimate tensile strength was too large in the fatigue simulation, this led to the decrease in the fatigue property and fatigue cycle number.

### 4.3. Effect of Notches width on Fatigue Property

**Figure 11:**
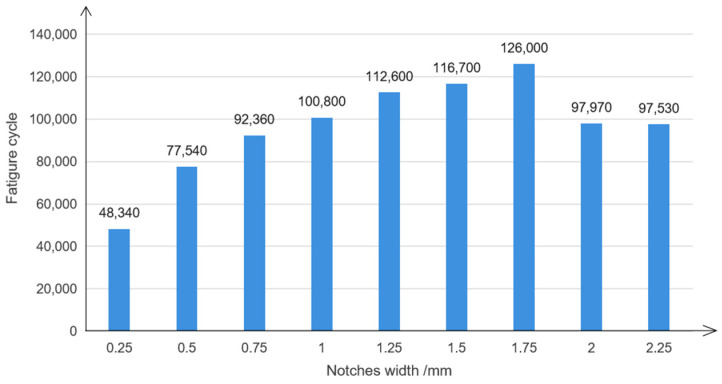
Effect of notches width on fatigue property.

Through the research on the influence of different notches longitudinal distance and notch length on fatigue property, it is known that the fatigue property of the specimens is the best when the notches’ longitudinal distance is 100 mm and the notches’ length is 1.5 mm. Therefore, under the condition that the notches’ longitudinal distance is 100 mm and the notches’ length is 1.5 mm, the influence of different notch widths on the fatigue property of the specimens as explored by changing the notches’ width, and the final simulation results are shown in Figure 11.

It can be seen from Figure 11 that with the increase in notches width, the fatigue cycle number first increased and then decreased. When the notches’ width was 1.75 mm, the number of fatigue cycles of the specimens reached a maximum value of 126,000 cycles, which is about 180% higher than that of the 0-0-type long fiber specimens without notches. When the notches’ width was less than 1.75 mm, with the increase in the notches’ width, the tensile strength of the specimens decreased in the effective range. The toughness increased, and the number of fatigue cycles increased. When the notches’ width was greater than 1.75 mm, the tensile strength of the specimens decreased rapidly with the increase in the notches’ width. The ratio of the stress to the ultimate tensile strength was too large in fatigue simulation, which led to the decrease in the fatigue property and fatigue cycle number.

### 4.4. Relationship between the Change in Strain Energy and Loading Slope

The displacements of 0-0-type and 10-2-type specimens were counted in one loading cycle. The ratio of tensile force to specimens’ cross-sectional area is the stress, and the ratio of displacement to specimens length is the strain. The specimens area and specimens length are fixed, so the relationship between tensile force and displacement is equal to the relationship between stress and strain. The loading stress level of this test material is the strain fatigue (alternating) loading within the yield limit. Therefore, this paper only discusses the relationship between the strain energy release rate and the slope of the loading line under the condition of linear elasticity. The tension–displacement relationships of 0-0-type and 10-2-type specimens are shown in Table 5.

**Figure 12:**
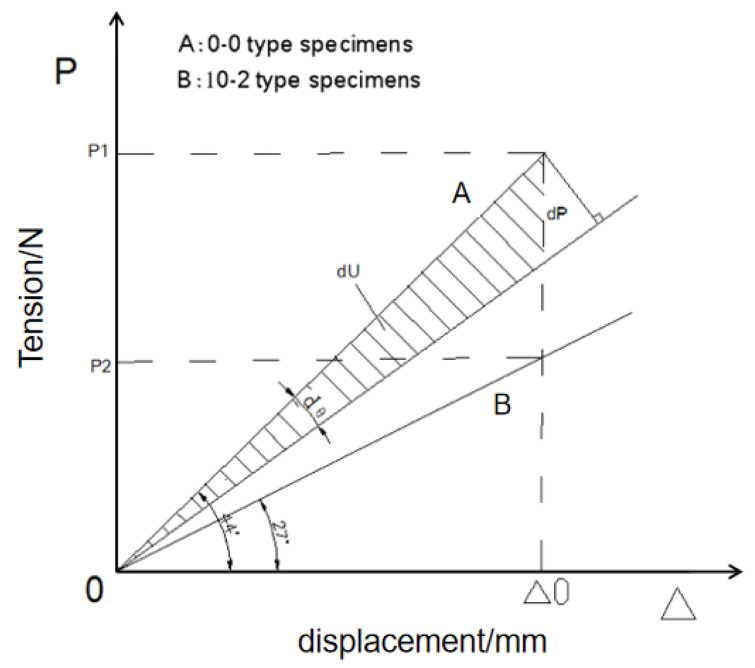
Schematic diagram of strain energy increment.

In order to compare the tension-displacement relationship and strain energy change rule of 0-0-type and 10-2-type specimens, and research on the causes of fatigue failure, the data in Table 4 and Table 5 are plotted as shown in Figure 12.

As can be seen from Figure 12,

(1) $$dU_{\Delta }=\frac{1}{2}\Delta \left(P_{2}-P_{1}\right)=\frac{1}{2}\Delta •dP$$

where *dU*_Δ_ is the increment of strain energy, Δ is the displacement, and *dP* is the load increment.

If the slope of the elastic loading line is m, the change rate of the slope relative to the load is:

(2) $${\left(dm\right)}_{\Delta }=\frac{P-dP}{\Delta }-\frac{P}{\Delta }=-\frac{1}{\Delta }dP$$

So:

(3) $${\left(\frac{dU}{dP}\right)}_{\Delta }=-\frac{1}{2}\Delta^{2}{\left(\frac{dm}{dP}\right)}_{\Delta }$$

Or:

(4) $${\left(\frac{dm}{dP}\right)}_{\Delta }=-\frac{2}{\Delta^{2}}{\left(\frac{dU}{dP}\right)}_{\Delta }$$

m=tanθ, so:

(5) $${\left(\frac{d}{dP}\tan\theta \right)}_{\Delta }=\sec^{2}\theta \frac{d\theta }{dP}=-\frac{2}{\Delta^{2}}{\left(\frac{dU}{dP}\right)}_{\Delta }$$

From trigonometric function, $\sec\theta =\left(\frac{\sqrt{P^{2}+\Delta^{2}}}{\Delta }\right)$. According to Figure 11, it can be seen that the microincrement *dθ* of the loading line bevel *θ* can be approximately expressed as:

(6) $$d\theta \approx \frac{\cos\theta }{\sqrt{P^{2}+\Delta^{2}}}dP=\frac{\Delta }{P^{2}+\Delta^{2}}dP$$

The results are as follows:

(7) $$\frac{d\theta }{dP}=\frac{\Delta }{P^{2}+\Delta^{2}}$$

By substituting Equation (7) into Equation (5), we can get the results:

(8) $${\left(dU\right)}_{\Delta }=-\frac{\Delta }{2}dP$$

Integral of the above formula:

(9) $$\Delta U=\int_{P_{2}}^{P_{1}}\left[-\frac{\Delta }{2}\right]dp=-\frac{\Delta }{2}(P_{1}-P_{2})$$

This is the difference in strain energy before and after the change in cross section under the condition of linear elasticity. When the cross section is reduced, the fatigue damage of the weak part of the material caused by the release of the excess strain energy can be avoided. The effect of strain energy on the fatigue of materials cannot be ignored.

Then, observe the change of slope of the tensile curve before and after of the change of cross section:

(10) $${\left(dU\right)}_{\Delta }=-\frac{1}{2}\Delta^{2}{\left(dm\right)}_{\Delta }$$

So:

(11) $$dm=-\frac{2}{\Delta^{2}}dU$$

The effect of the change of strain energy on the slope of loading line is expressed. In the linear elastic range, the above equation can be integrated:

(12) $$\Delta m=\int_{U_{2}}^{U_{1}}-\frac{2}{\Delta^{2}}dU=-\frac{2}{\Delta^{2}}(U_{1}-U_{2})=-\frac{2}{\Delta^{2}}\left(\Delta U\right)$$

where Δ*U* is the difference in strain energy.

The change in loading line slope is proportional to the change in strain energy in the linear elastic range. It can be seen from Figure 12 that the loading line slope of 0-0-type specimens is 44° and of 10-2-type specimens is 27°. The loading line slope of 0-0-type specimens is obviously larger than that of 10-2-type specimens. The area surrounded by the tensile curve and displacement axis of the 0-0 type specimens is larger than the 10-2-type specimens, and the strain energy released is larger. Strain energy constitutes the driving force for the destruction of molecular bonds, cracks, defects, and other weak parts of materials. It forms the source of fatigue in the weak parts of materials; then fatigue crack propagation occurs, and finally fracture failure occurs. Therefore, 0-0-type specimens are more prone to fatigue fracture, and its fatigue property is not as good as 10-2-type specimens.

The finite element analysis of the specimen was carried out by ANSYS, and the analysis results were imported into nCode to measure the fatigue cycles number of the specimens under different notch longitudinal distances, notch lengths, and notch widths. The results are as follows: in this experiment, when the notches’ longitudinal distance was 100 mm, the notches’ length was 1.5 mm, and the notches’ width was 1.75 mm, the fatigue cycles number of the specimens reached the maximum value of 126,000 cycles, which is about 180% higher than that of 0-0-type long fiber specimens without notches.

## 5. Conclusions

Through tensile test and fatigue life simulation of glass fiber-reinforced resin matrix composite laminates, the tensile strength of specimens with different fiber structures and fatigue cycles number under the same stress amplitude were measured. It seems that the following conclusions can be drawn:

(1) Compared with the continuous long-fiber specimens, the tensile strength of the specimens with local fiber notches decreases slightly. However, with the increase in notches’ longitudinal distance, the degree of influence of tensile strength decreases significantly.
(2) In this experiment, when the longitudinal distance of the notches was about 100 mm, the number of fatigue cycles of specimens reached the maximum value of 72,480 cycles, which is about 61% higher than that of 0-0-type long fiber specimens without notches. Additionally, the strength-fatigue comprehensive mechanical properties are better.
(3) In this experiment, under the condition that the notches’ longitudinal distance was 100 mm, when the length of the notches was 1.5 mm, the fatigue cycles number of the specimens reached the maximum value of 77,540 cycles, which was 72% higher than that of the 0-0-type long fiber specimens without notches.
(4) In the experimental conditions in which the notches’ longitudinal distance was 100 mm and the notches’ length was 1.5 mm, and the notches’ width was 1.75 mm, the number of fatigue cycles of the specimens reached the maximum value of 126,000 cycles, which is about 180% higher than that of the 0-0 long-fiber specimens without notches.

## Figures and Tables

**Table 1:** Mechanical properties of the material.

Parameter	Numerical Value
Young’s modulus/GPa	*E_x_* = 45, *E_y_* = *E_z_* = 15
Shear modulus/GPa	*E_yz_* = 4, *E_xy_* = *E_xz_* = 5
Poisson’s ratio	*ν_yz_* = 0.4, *ν_xy_* = *ν_xz_* = 0.3

Note: *x* is the direction along the length of the fibers in a monolayer plate. *y* is the vertical direction of the fiber length in in the monolayer plate. *z* is the perpendicular direction of the monolayer.

**Table 2:** Structural design of composite monolayers.

Model of Monolayers	Longitudinal Distance of Notched Fiber/mm	Lateral Distance of Notched Fiber/mm	Length of Notches/mm	Notches State of Two Adjacent Lines
0-0	0	0	0	No notches
4-2	40	10	2	Interlaced
6-2	60	10	2	Interlaced
8-1	80	10	2	Parallel
8-2	80	10	2	Interlaced

Note: the first number in the model of monolayer represents the longitudinal distance of notched fiber, and the unit is 10 mm. The second number represents the notched fiber state of two adjacent lines, 0 is no notches, 1 is parallel, 2 is interlaced.

**Table 3:** Tensile test results of specimens.

Model of Specimens	Average Value of Tensile Force/kN	Average Value of Tensile Strength/MPa
0-0	12.3	984.0
4-2	11.14	891.2
6-2	11.77	941.6
8-1	12.05	964.0
8-2	12.22	977.6

Note: the first number in the model of monolayer represents the longitudinal distance of notched fiber, and the unit is 10 mm. The second number represents the notched fiber state of two adjacent lines, 0 is no notches, 1 is parallel, and 2 is interlaced.

**Table 4:** Structural design of composite monolayers.

Model of Monolayers	Longitudinal Distance of Notched Fiber/mm	Lateral Distance of Notched Fiber/mm	Length of Notches/mm	Notches State of Two Adjacent Lines
9-2	90	10	2	Interlaced
10-2	100	10	2	Interlaced
11-2	110	10	2	Interlaced
12-2	120	10	2	Interlaced

Note: the first number in the model of monolayer represents the longitudinal distance of notched fiber, and the unit is 10 mm. The second number represents the notched fiber state of two adjacent lines; 0 is no notches, 1 is parallel, 2 is interlaced.

**Table 5:** Tension-displacement relationship of 0-0-type specimens.

Tension/N	Displacement/mm	
	0-0 Type	10-2 Type
738.0	0.764	1.448
2398.5	2.484	4.707
4059.0	4.203	7.966
5719.5	5.923	11.225
7380.0	7.640	14.480
